# The Emerging Role of Carbon-Ion Radiotherapy

**DOI:** 10.3389/fonc.2016.00140

**Published:** 2016-06-07

**Authors:** Daniel K. Ebner, Tadashi Kamada

**Affiliations:** ^1^Research Center for Charged Particle Therapy, National Institute of Radiological Sciences, Chiba, Japan

**Keywords:** particle beam therapy, carbon-ion radiotherapy, adaptation, performance, radioresistance

## Abstract

Carbon-ion radiotherapy (CIRT) has progressed rapidly in technological delivery, indications, and efficacy. Owing to a focused dose distribution in addition to high linear energy transfer and subsequently high relative biological effect, CIRT is uniquely able to target otherwise untreatable hypoxic and radioresistant disease while opening the door for substantially hypofractionated treatment of normal and radiosensitive disease. CIRT has increasingly garnered international attention and is nearing the tipping point for international adoption.

## Introduction

In 1952, the first human patients were treated by John Lawrence and Cornelius Tobias with helium and deuteron particle beams (1). Subsequently, interest in particle beams expanded, with proton facilities emerging throughout the world. However, as the biological impact of protons mirrored that of X-ray therapy, attention turned to heavier ions due to a higher biological impact owing to higher linear energy transfer (LET) (2). In 1975, with the installation of the BEVALAC to the Lawrence Berkeley Laboratory (LBL), extensive research into the clinical potential of heavy-ion beams more formally began (3).

In response to the initial successes at LBL, in 1984, the Japanese government began construction on the world’s first heavy-ion facility designated for medical use at the National Institute of Radiological Sciences (NIRS), staffing it with scientists returning from the BEVALAC and LBL. The Heavy Ion Medical Accelerator in Chiba (HIMAC) was completed in 1993, with clinical trials in carbon-ion radiotherapy (CIRT) beginning in June 1994.

Similar to the BEVALAC, the HIMAC provided for passive-beam irradiation. NIRS was alone in offering CIRT until 1997, when the GSI Carbon-Ion Radiotherapy Facility in Germany came into operation, pioneering raster scanning heavy-ion beams in clinical practice. GSI treated 440 patients with good results before its closure in 2008 (4). NIRS completed development of a pencil-beam raster scanning (PBS) treatment facility in 2012, and initial clinical trials are promising.

Developments in diagnostic technologies have enabled new therapeutic applications, such as markerless respiration-gated PBS irradiation. The enhanced radiobiological effect of the carbon-ion, concentrated and converged into a highly conformal dose distribution coinciding with target-respiratory movement, has allowed for medical care of radioresistant, previously untreatable disease (5–7). Further, these advantages have provided for hypofractionated radiotherapy of more common diseases, as well as improved adverse effect profiles, in comparison to conventional therapy. Altogether, this has lead to excellent treatment results in numerous diseases.

To date, nearly 70 protocols have been conducted at NIRS to delineate CIRT efficacy, safety, optimal treatment indications, and dose fractionation (8). Protocols begin with phase I dose-escalation studies focused on minimizing adverse effects. This is followed by phase II evaluation of treatment efficaciousness with longitudinal follow-up. If feasible, protocols exploring hypofractionation follow. Initial protocols began with low doses and an average of 18 fractions, but after critical review of technical and clinical data, today cases average 11–12 fractions. One or two total fractions are possible for indicated lung and liver disease, respectively. As such, the Hospital of the NIRS has reached a treatment capacity of between 900 and 1000 patients per year.

Carbon-ion radiotherapy facilities and faculty continue to grow in number and experience, with 8+ operational centers and over 15,000 patients treated to date (9). In Japan, in addition to the four heavy-ion radiotherapy facilities in operation prior to 2015, the Kanagawa Cancer Center’s carbon-ion facility began treatment in December 2015, and plans exist to construct facilities in Osaka City as well as Yamagata and Okinawa Prefectures. In light of the concentration of CIRT facilities in Japan, the Japan Carbon-ion Radiation Oncology Study Group (J-CROS) was organized to coordinate multi-institutional studies moving forward. Internationally, Austria will open a CIRT center in 2017, with centers in South Korea, Taiwan, China, and the United States in various states of development. Further, the clinical successor to the GSI Carbon-Ion Radiotherapy Facility, the Heidelberg Ion Therapy Center (HIT), has begun a number of randomized trials, testing carbon(-boost) versus other irradiation modalities (10–14).

In this paper, we aim to update on the expanding role of CIRT in cancer treatment as of 2016.

## Features of CIRT

**Figure 1:**
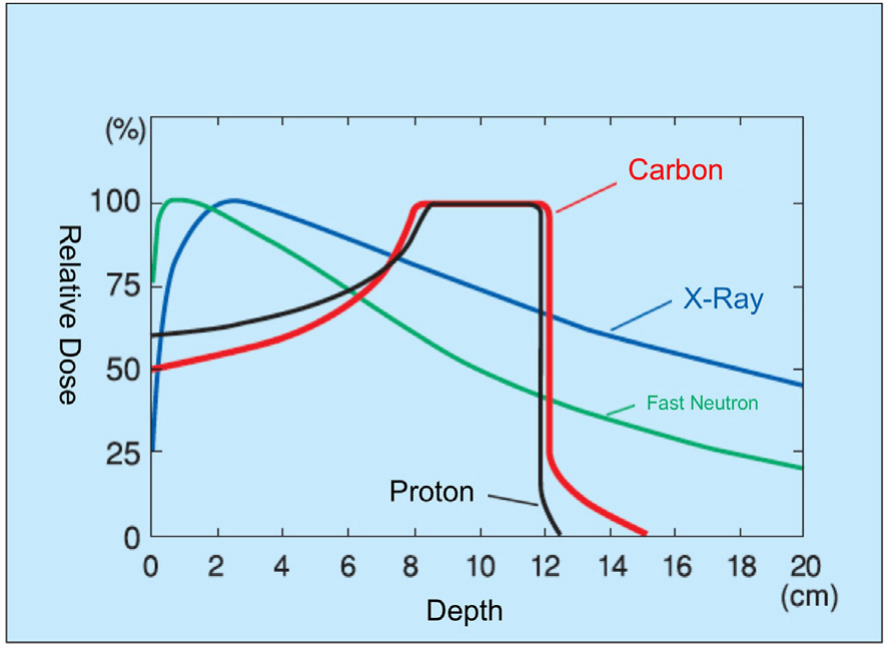
**Dose distribution of heavy-particle beams**.

In comparison with conventional radiotherapy, particle beams possess different physical and biological characteristics that must be weighed when considering treatment. While conventional radiation generally passes continually through a biological target, with dose delivered roughly equivalently throughout the beam path, particle beams release energy at the inverse of their velocity (Figure 1). Particle beams thus deliver a lower entry dose, depositing the majority of their energy at the flight path terminus, yielding an asymptotic dose peak (the “Bragg Peak”) (15). This allows for a dose concentration distribution impossible with conventional irradiation methods.

Today, proton dominates particle therapy. However, the larger mass of carbon results in decreased beam scattering, yielding a sharper dose distribution border with minimal penumbra (16). Radiobiologically, carbon-ion beams result in two to three times the relative biological effect (RBE; the biological effectiveness of one type of ionized radiation relative to another, given the same amount of absorbed energy) of proton and conventional irradiation methods (17). In comparison with photon therapy, CIRT does not show an oxygen effect, sublethal damage repair, and has less cell-cycle-related radiosensitivity.

These unique characteristics formed the rationale in initially applying carbon to radioresistant and/or hypoxic disease. Further indications then arose: the sharp dose distribution allows therapeutic dose delivery to disease juxtaposed with vital, radiosensitive organs (18–20). With radionormal or radiosensitive disease, short-term hypofractionated treatment becomes possible, owing to diminished dose delivered to healthy tissue.

## Carbon-Ion Radiotherapy Treatment

To date, over 9000 patients have undergone CIRT at NIRS, with 12,000 across all facilities in Japan and over 15,000 worldwide. In 2003, upon review of the first 9 years of NIRS’ clinical trials, the Japanese government allowed CIRT availability to the general public. CIRT has demonstrated efficacy against prostate, head and neck, lung, and liver cancers, bone and soft tissue sarcomas, locally recurrent rectal cancer, and pancreatic cancer, including locally advanced disease (8, 19, 21). Below, we provide a brief summary of the current most common indications and the data supporting their treatment.

At NIRS, over 2000 prostate cancer patients have been treated with CIRT, comprising approximately a fourth of CIRT-treated cases. Half of these cases are considered high risk at the time of treatment (determined by high PSA, T3 status, or high Gleason score). Initially, dose escalation in 20 fractions was performed, followed by investigation of hypofractionation. From 2007 to 2013, 781 patients were treated with 57.6 Gy (RBE) delivered in 16 fractions, with 5-year overall survival (OS) and biochemical relapse-free rates of 96.9 and 92.8%, respectively. No grade 3 or higher toxicity was seen. In 2014, treatment shifted to 12 fractions [51.2 Gy (RBE)] delivered over 3 weeks, yielding 100% cause-specific survival at a median follow-up of 32.3 months. At this dose-fractionation, no grade 3 or greater acute or late toxicities were observed, comparing favorably to conventional radiotherapy. Long-term data are pending, and further hypofractionation is being considered (22–24). Internationally, two randomized trials comparing proton and carbon are under recruitment at HIT (10).

Highly radioresistant non-squamous-cell carcinomas accounted for the majority of head and neck disease treated at NIRS, consisting of 11% of CIRT cases there. In a review of 240 patients (243 lesions), over a 9-year period, excellent results have been reported. 91% of patients received 57.6 Gy (RBE) with the remainder receiving 64.0 Gy (RBE), both delivered in 16 fractions. Approximately half of the high-dose group consisted of bone and soft tissue sarcomas of the head and neck. The 5-year local control (LC) rate was 68% across all head and neck cancers, with OS of 47% (LC/OS histological breakdown: 75/35% mucosal malignant melanoma, 73/68% adenoid cystic carcinoma (ACC), 73/56% adenocarcinoma, 24/36% sarcomas, 61/31% papillary adenocarcinoma, and 61/17% squamous cell carcinoma). Acute grade 3 skin and mucosal reactions were seen in 15 (6%) and 24 (10%) of patients, respectively, with no acute grade 4 or higher toxicity seen. No late skin grade 3 or greater toxicities were noted. Late mucosal side effects included no grade 3, but four cases of grade 4 ipsilateral blindness (25, 26). In 109 head-and-neck-based malignant mucoscal melanoma patients treated concomitantly with dacarbazine, nimustine, and vincristine (DAV), a 5-year LC rate of 82% with OS of 52% was achieved versus 33% OS with carbon alone (27). At HIT, carbon ions were used as boost in ACC, achieving 78% LC at 4 years, with rates of severe late toxicity <5% (28).

A majority of bone and soft tissue tumors are radioresistant and form a prototypical disease for CIRT treatment. Thus, despite being comparatively rare, these make up 11% of CIRT cases at NIRS. In particular, in both the skull base and trunk, chordoma, osteosarcoma, spinal tumors, and retroperitoneal tumors treated with CIRT have demonstrated satisfactory results (27, 29–33). Skull base and paracervical disease treated with 48.0–60.8 Gy (RBE) in 16 fractions yielded an overall LC and OS rate of 86 and 85%, respectively (LC/OS: 87/90% chordomas, 81/76% chondrosarcomas, 89/73% olfactory neuroblastomas, and 83/86% meningiomas). 24.5% of patients experienced grade 2 or greater radiation-induced brain injury (RIBI) (7.0% symptomatic), with a single case of grade 4 RIBI (27, 34, 35). This reinforced similar results from GSI, where LC of 70% at 5 years in chordoma and 87% at 4 years in chondrosarcomas, with limited toxicity, were achieved (36, 37). Randomized trials at HIT for these diseases are underway. In unresectable primary spinal sarcoma, following a dose of 52.8–70.4 Gy (RBE) in 16 fractions, 5-year LC and OS were 79 and 52%, respectively, with smaller disease (<100 cm^3^) demonstrating 100% LC. Three patients (6%) experienced a grade 3 or greater adverse effect, and seven experienced vertebral body compression (32). In unresectable retroperitoneal sarcoma, following dosing of 52.8–73.6 Gy (RBE) in 16 fractions, 5-year LC and OS was 69 and 50%, respectively. No grade 3 or greater toxicity was noted (33). In unresectable truncal osteosarcoma, following a median 70.4 Gy (RBE) applied in 16 fractions, LC of 62% and OS of 33% was seen, with no grade 3 or greater toxicity noted. Worse outcomes were seen in patients with a clinical target volume >500 cm^3^ (31). At HIT, locally unresectable osteosarcomas are treated with carbon and chemotherapy in an ongoing trial that includes the only cohort of CIRT-treated pediatric patients. Results are forthcoming (11, 12).

With lung and liver cancers, the improved dose distribution and strong RBE of CIRT led to prospective trials in hypofractionation, yielding excellent results (20, 38–40). Lung cancers encompass 11% of cases at NIRS, and currently, single-fraction delivery of 50 Gy (RBE) is indicated for Stage I, T1 and T2 non-small-cell disease. This has demonstrated a 5-year LC rate of 80.4% for patients receiving doses of 36.0 Gy (RBE) or more (T1: 86.0% and T2: 71.7%), with 5-year OS of 56.3%. For patients receiving 48 or 50 Gy (RBE), 2-year LC and OS were both 95% (39). The first non-Japanese lung cancer CIRT trial, at HIT, will be a prospective clinical trial on patients with chest wall infiltration (10). Hepatocellular carcinoma, making up 10% of CIRT indications, leads to notably poor survival rates due to inherent radiosensitivity of the liver combined with poor resectability (41). Current hypofractionation efforts led to a two-fraction regime consisting of 45.0 Gy (RBE). This has yielded OS and LC rates of 71 and 83% at 3 years, respectively. No grade 3+ reactions were noted in a 45 Gy (RBE) or higher dose treatment group (42). Of note, four-fraction, 52.8-Gy (RBE) treatment of tumors lying near to the porta hepatis has yielded good control: 5-year LC of 87.8% with OS of 22.2% without similar side-effect profiles to non-porta hepatis cases (20). The PROMETHEUS-1 trial at HIT reported initial results in 2013: at 11 months, LC of 100% was achieved with no severe adverse events reported (43).

Locally recurrent rectal cancer (5% of cases), pancreatic cancer (4% of cases), and cervical adenocarcinoma and related cancers (gynecological tumors encompass 3% of cases) all demonstrate degrees of radioresistance, but CIRT has demonstrated excellent performance in treating these diseases. A phase I/II dose-escalation study of 170 recurrent rectal cancer patients was performed at NIRS, with escalating dose between 67.2 and 73.6 Gy (RBE) delivered over 16 fractions in 4 weeks. LC at 3 years was 92% for 73.6 Gy (RBE), with OS of 59% at 73.6 Gy (RBE) at 5 years. No acute grade 3 or greater adverse events were seen, with two grade 3 late skin and one grade 3 late gastrointestinal reaction noted (44, 45). The forthcoming PANDORA-01 trial at HIT will further evaluate use of carbon in the setting of recurrent rectal cancer (13). The results for locally advanced pancreatic cancer have drawn international attention with combined CIRT-gemcitabine therapy, yielding a 1- and 2-year freedom from local progression rate (FFLP), evaluated by ^18^FDG uptake, of 63 and 30%, with OS at 1 and 2 years of 73 and 35%, respectively. When limited to Stage III disease, 2-year FFLP and OS improved to 40 and 48%, respectively. 53% of patients experienced grade 3–4 hematological toxicity, and 7% experienced grade 3 anorexia. One case (1%) of grade 3 intratumoral infection was noted. None of these reactions were life-threatening (21). The forthcoming PHOENIX-01 trial at HIT will evaluate advanced pancreatic cancer treatment with scanning carbon-ion beam irradiation in combination with gemcitabine (14). With regard to cervical cancer, 58 locally advanced adenocarcinoma cases were treated in a dose-escalation study [62.4–74.4 Gy (RBE) in 20 fractions] between 1998 and 2010, with 5-year LC of 54.5% and OS of 38.1%. One patient experienced a grade 4 rectal complication, with no other grade 3 or higher toxicities reported (46).

Radiotherapeutic treatment of brain malignancies remains a substantial challenge. Combs and colleagues conducted a pooled analysis of HIT and Japanese data regarding the usage of carbon-ion boost (CIB) in the treatment of anaplastic astrocytoma (AA) and glioblastoma (GBM) (47–49). Postoperatively, 50-Gy photon with nimustine hydrochloride was administered, with 16.8–24.8 Gy (RBE) CIRT provided as boost. In GBM and AA, median OS was 18 and 35 months with CIRT versus 14 and 39 months with standard postoperative radiochemotherapy (RCT) with temozolomide. Progression-free survival of GBM and AA were 6 and 6 months (RCT) and 8 and 34 months (CIRT), respectively. The potential benefit of CIRT noted is under further evaluation in the CLEOPATRA trial at HIT (47).

Overall, CIRT has demonstrated good adaptability for difficult-to-treat, radioresistant disease, while allowing accelerated, hypofractionated treatment of other disease. Distant metastasis remains a challenge, but initial evaluations of CIRT concurrent with chemotherapy has demonstrated satisfactory performance (21, 27).

## Future Directions

The future of charged particle therapy as of 2016 appears bright, with implementation of respiration-gated fast PBS (50), markerless tracking (51), a range-shifter-free multiple-energy modulation system, and completion of the second carbon-ion rotating gantry in the world at NIRS, following the first at Heidelberg. Nine plus new CIRT centers are opening worldwide. However, the high cost associated with the construction, maintenance, and operation of CIRT facilities, as well as the corresponding costs in staff development and support, presents a challenge for extension of the technology outside of the developed world.

As such, a great deal of work remains. Development in cost-saving and improved miniaturization of existing technology is necessary. To date, these efforts have produced CIRT accelerator and synchrotrons at one-third the cost and size of the original HIMAC, which are in operation at the Saga-HIMAT, Gunma University, and Kanagawa Cancer Center i-ROCK facilities. Superconduction technology allowed for the recently completed rotating gantry at NIRS to be built with a length and diameter of 13 and 5.5 m, respectively, versus the existing gantry at HIT, which is 25 m × 13 m (52). Ongoing development aims to further employ superconducting technology in the accelerator and overall device, producing a total CIRT setup dubbed the “Super MINIMAC” that will fit within 20 m^2^. Meanwhile, limited research has been published on the cost-efficacy of CIRT (53–55), which would appear to improve with each new technological development; focused evaluation may be necessary to facilitate international development.

In Japan, CIRT is available as a private treatment to the general public. Discussion to extend national insurance coverage to CIRT is ongoing. However, despite the current cost burden for patients (3.2 million yen/~28,000 USD for a treatment course, regardless of fractionation; in Germany, treatment costs ~1000 Euro per fraction), the number of patients from both within and outside Japan continues to increase.

Clinically, as the majority of cases treated with CIRT in the world were treated at NIRS, the majority of available clinical data is focused at a single institution spread over 20 years (19). As center numbers increase, multi-institutional trials and randomized, internationally coordinated trials may begin.

An international ecosystem supporting and interweaving CIRT clinical, physical, and biological development is also necessary. It is known that the LET of particle beams are non-homogenous throughout the irradiated region, yielding variations in RBE (56). As carbon is a high LET beam, these variations are more appreciable than with low LET proton irradiation. Due to a risk of consequent under- or over-treatment and toxicity, complex dosing models are required in the use of heavy ions. Of particular note, the RBE model varies between international institutions. Within Japan, the MKM2010 model, a revision of the Microdosimetric Kinetic Model, has been developed and implemented (57). In Europe, versions of the local effect model (LEM) are dominant. Efforts to improve international standardization are progressing, with work by Fossati and colleagues providing for MKM2010 dose translation to the LEM model and vice versa (58). Improved model accuracy and careful manipulation of the high LET/RBE regions may enable LET painting of tumors (59). This “intensity modulated carbon therapy” may further improve dose distribution, and research to this end is underway.

## Conclusion

Since 1970, heavy-ion radiotherapy has progressed rapidly in technological delivery and, consequently, in indications and efficacy. The ability for the carbon-ion beam to offer short-term, minimally invasive, function-, tissue-, and form-sparing treatment has garnered international attention, with the technology nearing the tipping point for international adoption. Technically, enhanced international collaboration is needed to produce an intercenter translatable dosing model consensus and to enhance results at the common borders between radiobiology and particle physics. Societally, cost and access to treatment remains a challenge, particularly in developing countries. However, evidence continues to mount for the superiority of carbon in the treatment of radioresistant, hypoxic disease. Coupled with the opportunity for substantially abbreviated treatment of common disease, carbon-ion radiotherapy looks increasingly appealing as a treatment modality deserving worldwide availability.

## Author Contributions

DKE and TK wrote and edited the manuscript.

## Conflict of Interest Statement

The authors declare that the research was conducted in the absence of any commercial or financial relationships that could be construed as a potential conflict of interest.
